# A hybrid approach to forecast the COVID-19 epidemic trend

**DOI:** 10.1371/journal.pone.0256971

**Published:** 2021-10-04

**Authors:** Saqib Ali Nawaz, Jingbing Li, Uzair Aslam Bhatti, Sibghat Ullah Bazai, Asmat Zafar, Mughair Aslam Bhatti, Anum Mehmood, Qurat ul Ain, Muhammad Usman Shoukat

**Affiliations:** 1 College of Information and Communication Engineering, Hainan University, Haikou, China; 2 State Key Laboratory of Marine Resource Utilization in the South China Sea, Hainan University, Haikou, China; 3 School of Geography (Remote Sensing), Nanjing Normal University, Nanjing, Jiangsu, China; 4 Department of Computer Engineering, BUITEMS, Quetta, Pakistan; 5 General Nursing College DHQ Hospital, Jhang, Pakistan; 6 Medical School of Southeast University, Nanjing, P.R. China; 7 School of Automation and Information, Sichuan University of Science and Engineering, Yibin, China; South China University of Technology, CHINA

## Abstract

Studying the progress and trend of the novel coronavirus pneumonia (COVID-19) transmission mode will help effectively curb its spread. Some commonly used infectious disease prediction models are introduced. The hybrid model is proposed, which overcomes the disadvantages of the logistic model’s inability to predict the number of confirmed diagnoses and the drawbacks of too many tuning parameters of the SEIR (Susceptible, Exposed, Infectious, Recovered) model. The realization and superiority of the prediction of the proposed model are proven through experiments. At the same time, the influence of different initial values of the parameters that need to be debugged on the hybrid model is further studied, and the mean error is used to quantify the prediction effect. By forecasting epidemic size and peak time and simulating the effects of public health interventions, this paper aims to clarify the transmission dynamics of COVID-19 and recommend operation suggestions to slow down the epidemic. It is suggested that the quick detection of cases, sufficient implementation of quarantine and public self-protection behaviours are critical to slow down the epidemic.

## 1. Introduction

Infectious diseases are diseases caused by various pathogens that can spread from person to person, animal to animal, or person to animal [1]. Most of the pathogens are microorganisms, and a small part are parasites. The ones caused by parasites are also called parasitic diseases. The epidemic prevention department must keep abreast of their incidence and take timely countermeasures [2]. Therefore, they should promptly report to the local epidemic prevention department within the prescribed time after they are discovered. An infectious disease transmitted from one person or other species to another person or species through various channels is called a legal infectious disease. Usually, this disease can be spread via air, water and food [3].

Whenever an epidemic of infectious diseases breaks out, the people in that region are severely affected economically and physically. For infectious diseases that no vaccine has been developed for, such as COVID-19, it cannot be treated with vaccines as the vaccination process is still under trials. The amount of vaccine availability is limited. So, predicting and preventing is the top priority to tackle the spread of Covid-19 [4]. A new type of coronavirus caused the 2019 coronavirus disease. On 11 February 2020, the World Health Organization Director General, Dr Tedros Adhanom, officially named the pneumonia caused by the novel coronavirus COVID-19 [5]. The main transmission route of COVID-19 is through respiratory droplets and human contact. Its incubation period is 1–14 days, while most infectious diseases are 14–28 days. During its incubation period, COVID-19 is contagious and usually spreads among people. After the patient is infected, there will be fever, the main symptom is a dry cough, and the patient will feel fatigued. A few infected people will also have nasal congestion, a runny nose and sore throat. Symptoms can include diarrhoea and severe disease [3]. Infected persons with severe onset will have restricted breathing or reduced oxygen content in the blood 7 days after onset. Since December 2019, the sudden emergence of the coronavirus epidemic has had a serious impact on the people of our country and the world and caused widespread concern from all walks of life. Pakistan is faltering under the influence of a double challenge, namely, the COVID-19 pandemic and the subsequent economic difficulties. This has created one of the worst stages in the country. Pakistan has been in a severe economic crisis. The first two cases of COVID-19 were reported in Pakistan on 26 February 2020, as announced by the prime minister’s special assistant on health [6]. From 20 February 2020 to 21 April 2021, Pakistan reported 772,381 cases of coronavirus with 16,600 deaths, 83,162 active cases and 672,619 recoveries. The overall recovery rate is 97.52%, while the death rate is 2.41%, and new cases are still increasing daily. To sum up, the 2019 coronavirus epidemic situation is critical and has a serious impact on the lives of the people in our country. In the absence of a vaccine, it cannot be fundamentally developed. To solve the pandemic issue, research on the disease’s prevention and prediction is vital. Despite the broadening COVID-19 impacts, there remain strategic public health questions. How many people will be infected in the next few months in order for the government to prepare for additional hospital availability? How does the situation change day by day? Can we predict/forecast the future numbers of people infected with COVID-19 by using daily updated data on the pandemic’s trajectory? These questions could be answered by forecasting the possible future of this pandemic through time-series models. However, widely used time-series models and tools do not necessarily have high prediction accuracy, especially for medical studies.

Regarding the prevention and prediction of the 2019 coronavirus, there are currently many prediction models, including the prediction model based on logistic regression analysis, the exponential growth curve prediction model, the long short-term memory (LSTM) prediction model, the autoregressive integrated moving average (ARIMA) model, the trigonometric exponential smoothing state-space model with boxcox transformation (TBATS) models [7], the back propagation algorithm (BP) neural network model [8, 9], and many more. In the current research, the dynamic model is used to predict the epidemic situation. Since the beginning of the epidemic, relevant research teams have modelled and analysed the new coronavirus. Li et al. [10] and others [11, 12] have modelled the new coronavirus forecasting, conducted research on data from Hubei Province and Wuhan City in China, and analysed the differences. The number of days in the incubation period affects the number of infections and the time when the inflexion point of the epidemic comes. Perrotta et al. [13] studied the impact of national protective measures on the spread of the virus. They compared the changing trends of suspension of work and school without restrictions on travel and restrictions on travel on susceptible, latent, infected and removed persons. Hao X et al. [14] and others [15, 16] used two model laboratory cases for the estimation of the overall COVID-19 spread situation. According to those model’s, the actual cases can be monitored using the total registered cases in hospitals, and forecasting will be effective if that data is utilised to estimate future results. Shen et al. [17] used a general nonlinear least-squares (NLS) growth model to fit the number of confirmed diagnoses in three stages. They found that the number of confirmed diagnoses experienced an exponential increase in the initial period (15 January to 27 January). After the sub-exponential growth in the middle period (27 January to 6 February), the confirmed diagnoses entered a sub-linear growth phase from 6 February until March 2020. Liu et al. [18] and others [19, 20] predicted the basic reproductive number of pneumonia infected by Wuhan and estimated that the basic reproductive number of COVID-19 was between 2.8 and 3.3. With the development of the COVID-19 epidemic and the continuous strengthening of prevention and control strategies, the transmission mechanism of the new type of coronavirus pneumonia requires further research [21, 22]. Syage et al. [23] used the SEIR dynamic model with the asymmetric gaussian model to predict the coronavirus epidemic in the USA. For those infected with the disease and those found to be infected, because it considers the transmission law of the 2020 coronavirus disease and the implementation of isolation measures, the asymmetric gaussian model can predict the development trend of the disease accurately [24]. At the same time, the traditional dynamic susceptibility-infection-recovery (SIR) model has also been widely used in the study of the epidemic trend of coronavirus disease in 2019 [25, 26]. Cooper et al. [26] carefully discussed the susceptible-infected-removed (SIR) model and its impact on the 2019 coronavirus disease epidemic. Results from that study reveal that the SIR model can provide us with insights and predictions of the spread of the virus in communities that the recorded data alone cannot. The SIR modelling approach deduced that the spread of COVID-19 could be under control in all communities considered if reasonable restrictions and strong policies are implemented to control the infection rates early on. SIR can consider factors, such as the transmission speed and mode of the infectious disease. However, since the various parameters cannot be comprehensive, the parameters cannot be dynamically changed at different epidemic stages. In this context, this study applies the improved SEIR model to the 2019 coronavirus epidemic prediction. It researches dataset of Pakistan because new cases in Pakistan are increasing daily and need to be modelled urgently to improve and cure the health system. In the traditional SEIR model, a new parameter, the infection rate of the exposed person to be infected by the susceptible population, is introduced, and the model is segmented according to the relevant urban migration model index. This paper combines the logistics growth model with the improved SEIR model to predict the 2019 coronavirus epidemic in Pakistan. With the goal of higher-precision prediction of the 2019 coronavirus epidemic, a visual system for forecasting the 2019 coronavirus disease epidemic will be built to provide a reference for the prevention and early warning of the 2019 coronavirus disease epidemic. Therefore, this study aims to discover how to scientifically and efficiently control the epidemic, analyse the early epidemic data in Pakistan, mathematically model the existing data and then predict the future development trend of the epidemic and provide theoretical support practical guidance for epidemic prevention and control.

The remainder of the paper is organised as follows. In Section II, we discuss the different models used in this study. Experimental results were discussed in Section III with the implementation of the proposed model. In Section IV, we present a systematic discussion and comparison of the work using other approaches. Finally, we draw conclusions in Section V.

## 2. Literature review and related models

This section discusses different models that are useful for forecasting Covid-19 and use practical medical terms for prediction.

### A. Severe Acute Respiratory Syndrome (SARS) propagation model

The spreading law of COVID-19 can be studied in conjunction with the predictive models commonly used in other infectious diseases in recent years. According to the statistics of SARS in Hong Kong and Singapore in 2003, a dynamic mathematical model has been established by research [27]. Chowell et al. [28] established a system dynamics model to study the importance of ’early detection, early isolation, and early treatment’ measures for controlling the spread of SARS disease. Another study further improved the model and proposed the SARS spatio-temporal propagation dynamics model using intelligent data analysis and a decision tree, which is closely related to the SARS model, and realised a series of rules to guide the progress of the virus [29].

### B. Logistic model

The logistic model, which was initially used to simulate the growth of the human population [30], is often used to simulate the growth law of the number of infectious patients, and its function expression is

$$P(t)=KP_{0}e^{rt}/[K+P_{0}(e^{rt}-1)]$$ (1)

where *P*_0_ is the initial population value, representing the initial number of infections in the infectious disease model;

*K* is the environmental capacity, which represents the maximum cumulative number of infections in the infectious disease model;

*r* is the measurement curve change rate, which represents the disease infection rate in the infectious disease model;

T is time;

*P*(t) is the population that changes with time, which represents the cumulative number of infections that change with time in the infectious disease model.

Because the model is monotonically increasing, it can only predict the cumulative number of infections but cannot predict the current number of confirmed cases.

### C. Prediction models for confirmed cases

Commonly used models for predicting the number of confirmed diagnoses include the susceptible-infectious (SI) model [31], the success-infectious-success-success-success-type (SIS) model [32], and the susceptible-infected-recovered (SIR) model [33], the SEIR model [34] and more. The complexity of these models increases in turn, and the complex models are more in line with the actual spread of infectious diseases. Even now, the SIR model is still advancing and is widely used. The traditional SIR model further introduces the patient’s rehabilitation process because the previous SI model only considered the outbreak and spread of infectious diseases. The SIR model divides the total population into three categories based on infectious diseases: susceptibles, or S category, refers to people who are not ill but are easily infected; infectives, or I category, refers to those infected and transmit to other people; recovered, or R category, refers to people who have been removed and recovered or died, but it should be noted that here the second infection after recovery is not considered. SIR is based on the following three assumptions:

1. The total population is always the same. The impact of natural death and natural birth on the population is not considered.
2. In the model, if a susceptible person encounters an infected person, they will be infected and gain infectious power. Furthermore, during a specific period, the infectivity of an infected person is proportional to the susceptible person in the total population.
3. At a specified time, the number of people who have been transformed into recovery is proportional to the number of people who have been infected.

If a particular type of infectious disease has an incubation period, then the SIR model does not meet the infectious characteristics of the disease [35]. The SEIR model appears, which introduces the exposed person, the E-type population, based on the SIR model. The total number of people is divided into four groups: S, E, I and R. SEIR is also based on the three assumptions of the SIR model. Moreover, SEIR added conditions based on the first three assumptions: susceptible people who have been in contact with the sick will not get the disease immediately but will become carriers of the pathogen of the disease and are classified as E-groups. The advantages and disadvantages of SEIR are as follows:

- Compared with the SIR model characteristic that the person is contagious by contact, the SEIR model considers that only some people susceptible to contact with the infected person are infectious. This characteristic makes the disease propagation period longer.
- The SEIR model does not consider more detailed factors in the epidemic prediction process, such as the degree of government control, the speed and scope of the flow of people, the psychological quality of the population, the physical characteristics of different people and the severity of symptoms of patients. These factors all impact the disease’s infection rate, the length of the infection period, and the number of infected contacts.
- It does not consider the various deviations among the various groups of people in the actual situation. For example, whether exposed people can also infect susceptible people, whether the mortality rate of different groups is the same and whether the recovered people will be infected again.

**Fig 1:**
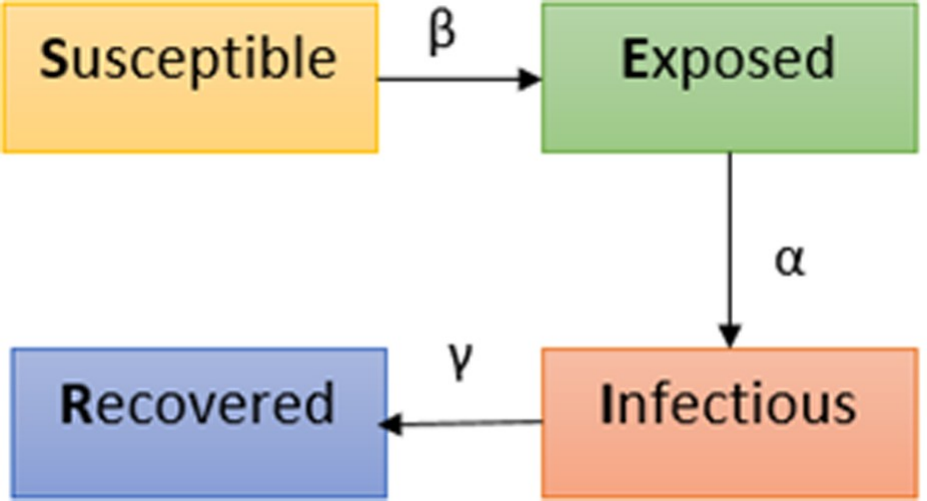
Schematic diagram of SEIR model.

Given these shortcomings, the model can be further improved. The schematic diagram of the most complex SEIR model is shown in Fig 1.

The SEIR model divides the population into four categories: Susceptibles, that is, healthy people without antibodies, and *S* is the number of susceptibles; Exposed, *E* is the number of latent; Infected, *I* represents the number of patients; recovery population (recovered), use *R* to represent the number of recovered patients (recovered people here include dead people). The total number of people *N* = *S* + *E* + *I* + *R*. The ratio of healthy people is *S/N*.

This model is more in line with the occurrence of COVID-19 than the other three models. The differential equation of the SEIR model is

dS/dt=−rβIS/N (2)

dE/dt=−rβIS/N−αE (3)

dI/dt=αE−γI (4)

dR/dt=γI (5)

where *r* represents the number of people encountered by an infected person, β is the infection rate probability of a healthy person becoming an exposed person, γ is the patient’s recovery rate and α represents the probability that an exposed person becomes a patient. The SEIR model is the most complicated of the four models, including the other three discussed before. It is also the most consistent with the actual spread of infectious diseases.

## 3. Proposed model

### Logistic and SEIR hybrid model

Among the above models, the logistic model can only predict the cumulative number of infections but cannot predict the current number of confirmed cases. The SEIR model can simulate the changes in the number of various populations during the spread of infectious diseases. However, suppose the current number (I) of confirmed diagnoses of COVID-19 per day is predicted, the number of healthy people (*S*), exposed people (*E*) and infected people (*I*) need to be calculated. In addition, the number of recovered people (*R*), the infection rate β of healthy people becoming latent, the infection rate of exposed people becoming infected α and the recovery rate of infected people becoming recovered γ need to be considered. Thus, a total of 7 parameters are adjusted. This process is complicated, and additional prior knowledge is required. Combining the logistic and SEIR model proposed in this paper (hybrid model) can solve these problems and significantly reduce tuning parameters. Only simple tuning of three parameters can achieve good prediction results.

The idea of the SEIR combined with the logistic model is the following: the parameters of the logistic model are the initial number of infections *P*_0_, the final cumulative number of infections *K* and the infection rate *r*, which can be automatically obtained after dataset training. We can use *r* in the logistic model to initialise β in the SEIR model. Using *P*_0_ in the logistic model initialises the initial number of infected persons (*I*) and the exposed number (*E*) in the SEIR model (or initialises *I* and *E* with the number of infected persons on the first day in the actual data). The number of rehabilitated persons (*R*) is initialised with the number of rehabilitated persons on the first day plus the number of dead persons in the actual data (or initialised to 0). Because the initial number of healthy people within a specific range is similar or more than the final cumulative number of infections, the number of healthy people in the SEIR model (*S*) can be initialised by multiplying *K* in the Logistic model by a coefficient greater than 1. The infection rate α of the exposed person becoming infected and the recovery rate γ of the infected person recovering can be adjusted relatively easily. The prediction effect will be improved.

Logistic_SEIR model implementation process and algorithm steps:

- Two datasets are used for training and testing purpose with a ratio of 8 (training): 2 (testing)
- Logistic function as defined in Eq (1) (Logistic formula): logistic_function (*t*, *K*, *P*_0_, *r*):
  $$return=KP_{0}e^{rt}/[K+P_{0}(e^{rt}-1)]$$
- Define the SEIR model function according to Eqs (2) ~ (5) (SEIR model differential equation):
  $$\begin{array}{c} diff\_SEIR\;\left(V=\left[S,E,I,R\right]\right):\\ Y[0]=-\beta \times V[0]\times V[2]\\ Y[1]=\beta \times V[0]\times V[2]-V[1]\times \alpha \\ Y[2]=\alpha \times V[1]-\gamma \times V[2]\\ Y[3]=\gamma \times V[2] \end{array}$$
  Return *Y*
- Using the nonlinear least-squares method to fit the logistic function on the training set, and get the parameters in the logistic model: the initial number of infected people *P*_0_, the final cumulative number of infected people *K*, the rate of infection *r*.
- Use the parameters obtained in the previous step to partially initialise the SEIR model parameters and initialise the remaining parameters.
- Through Matlab, we solve the differential equation of the SEIR model and obtain the predicted value of the number of confirmed patients.

The hybrid model proposed in this paper dramatically reduces the number and difficulty of tuning a single SEIR model parameter from 7 to 4. The parameters that need tuning can be adjusted within a specific range, making it easier to obtain good prediction results.

## 4. Methodology

Experimental data has been taken from the Pakistan COVID website (https://covid.gov.pk/) [36]. The data dates run from March 2020 until April 2021. Data includes date, infected person, suspected person, death, cure and more. To calculate the current number of confirmed cases, the number of confirmed cases is equal to the number of infected people minus the number of deaths minus the number of people cured. During training and prediction, the date is used as the independent variable *x* and the number of existing confirmed diagnoses is used as the dependent variable *y*. For convenience, the date is changed; if the infected person first appeared on 01 March 2020 is recorded as 0, and the subsequent dates are extended by 1, 2, 3, and so on.

**Fig 2:**
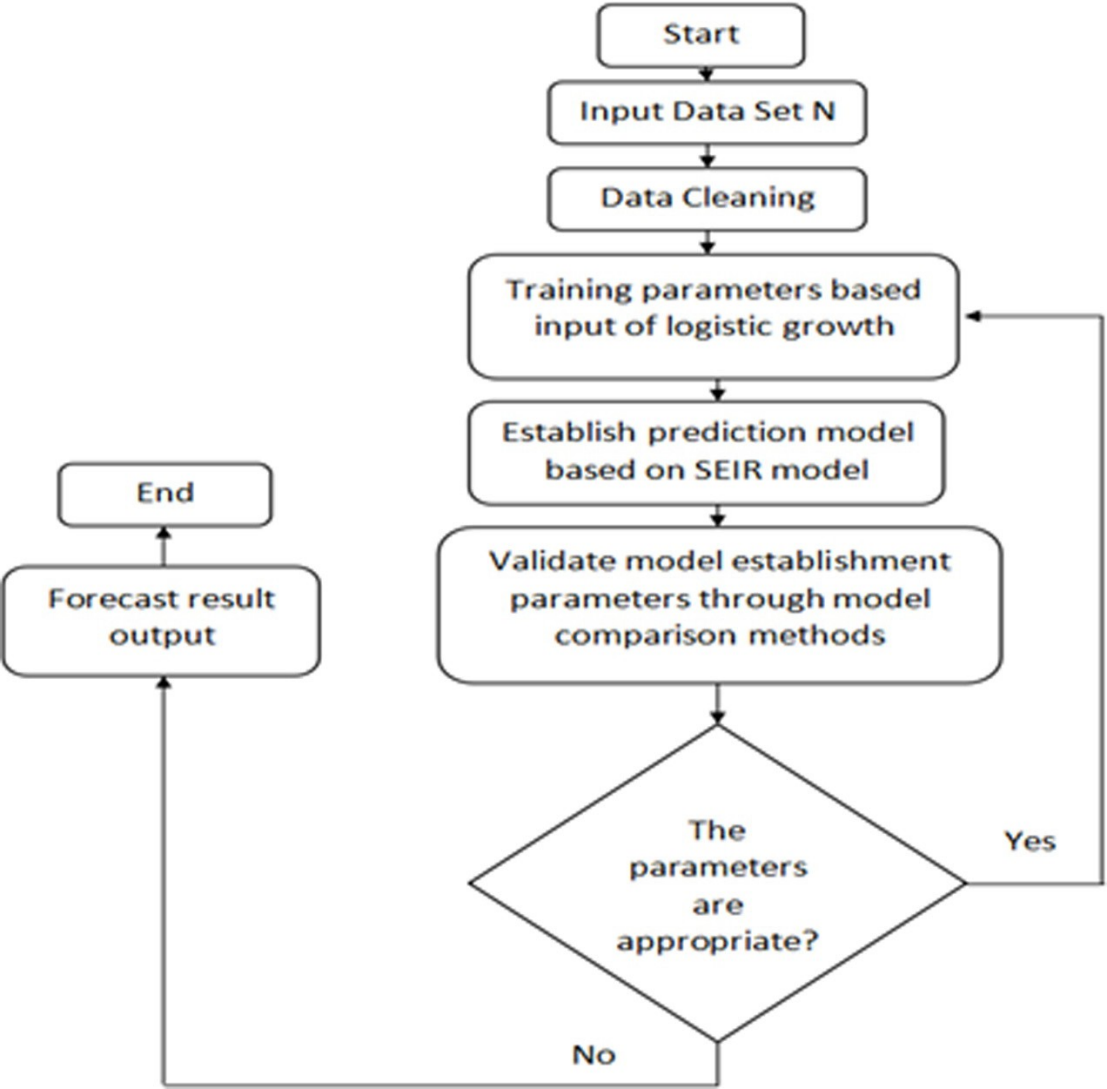
Flowchart of study.

The testing environment used CPU (Windows 10, Core i7) and Matlab with different libraries installed. Fig 2 shows the implementation methodology of the system.

The experiment is divided into two parts: 1) our hybrid model is used to predict the number of confirmed diagnoses; 2) different factors (exposed rate and infectious rate) which impact results are studied.

### A. Prediction of cases

Define the logistic function according to the logistic formula and fit the logistic function to the training set using the nonlinear least-squares method to obtain the values of the three parameters of the initial number of infections *P*_0_, the final cumulative number of infections *K* and the rate of infection *r*. Use the trained parameters *K*, *P*_0_ and *r* to initialise parameters of the SEIR model. In the SEIR model, *S*_0_ represents the initial number of healthy people and the parameter *K* in the logistic model represents the environmental capacity. Considering that the initial healthy people are more than the final cumulative number of infected people, this study uses *K* multiplied by a coefficient greater than 1 to calculate *S*_0_ for initialisation.

*E*0 represents the initial number of exposed persons in the SEIR model, initialised with the parameter *P*_0_ in the logistic model. *I*_0_ in the SEIR model is initialised with the number of infections on the first day in the data set, and *R*_0_ in the SEIR model is initialised with the number of deaths and recovery on the first day of real data. Since the exposed person can also infect, the infection rate is divided into the infection rate of the exposed person β_2_ and the infection rate β of the infected person. In this paper, the value of the two infection rates β_2_ and β multiplied by *r* divided by *N* is initialised with *r/N* in the logistic model parameters. The infection rate α from the exposed person to the infected person is initialised with 1/4 (the probability of the exposed person becoming positive α is large, so this article assigns α to the reciprocal of 1 to 6, and the comparison shows that α = 0.25 has the best fitting effect). The recovery rate γ is initialised with 0.05 (the cure rate is constantly changing with the change in the epidemic situation). Different studies used different parameters and have different results according to their country’s conditions.

Tables 1 and 2 show that our values are slightly changed because of the better results on new values as γ, in other studies, reflect the values of 0.07, but in our case, it gives better results on the value of 0.05. Therefore, we compare γ with different values and compare between 1/20 and 1/2 to find that γ = 1/18 fits the training set, and the prediction effect is the best on the test set. As the epidemic develops, the rate increases, so longer-term predictions need to increase the recovery rate further). To illustrate the effect of the model in this paper, a separate SEIR model is used as a contrast experiment. The initialisation of the individual SEIR model parameters must be based on additional data, making it more complicated. According to the existing data, the cumulative peak number of infected people is estimated to be more than 95,000.

**Table 1:** SEIR Parameters initialisation.

Parameters	Value used for this analysis	Source
Susceptible (S)	S = 25,000,000	[37]
	S rate = 25,000,000/32,000,000	
	= 0.78125	
Exposed (E)	E = 25, 717	[38]
	E rate = 25, 717/32,000,000	
	= 0.0008	
Infected (I)	I = 1608	[38]
	I rate = 1608/32,000,000	
	= 0.00005	
Recovered/died (R)	R = 216	[35]
	R rate = 216/32,000,000	
	= 0.000006	

**Table 2:** SEIR parameters initialisation.

Coefficients	Value used for this analysis	Source
β = coefficient of infection	β = β_0_ * k	[39, 40]
	β = coefficient of infection rate (R)	
	β_0_ = probability of infection per exposure (R_0_)	
	k = frequency of exposure	
	β^1^ = 6.47	
	β^2^ = 1.05	
σ = coefficient of latency	T_e_ = average latency	[41]
	= 5.2 days	
	σ = 1/ T_e_	
	= 1/5.2	
	= 0.19	
γ = coefficient of migration rate	ɣ = 1/ T_i_	[42]
	T_i_ = average recovery time	
	T_i_ = 14.5 days	
	γ = 1/ T_i_	
	= 0.07	

**Fig 3:**
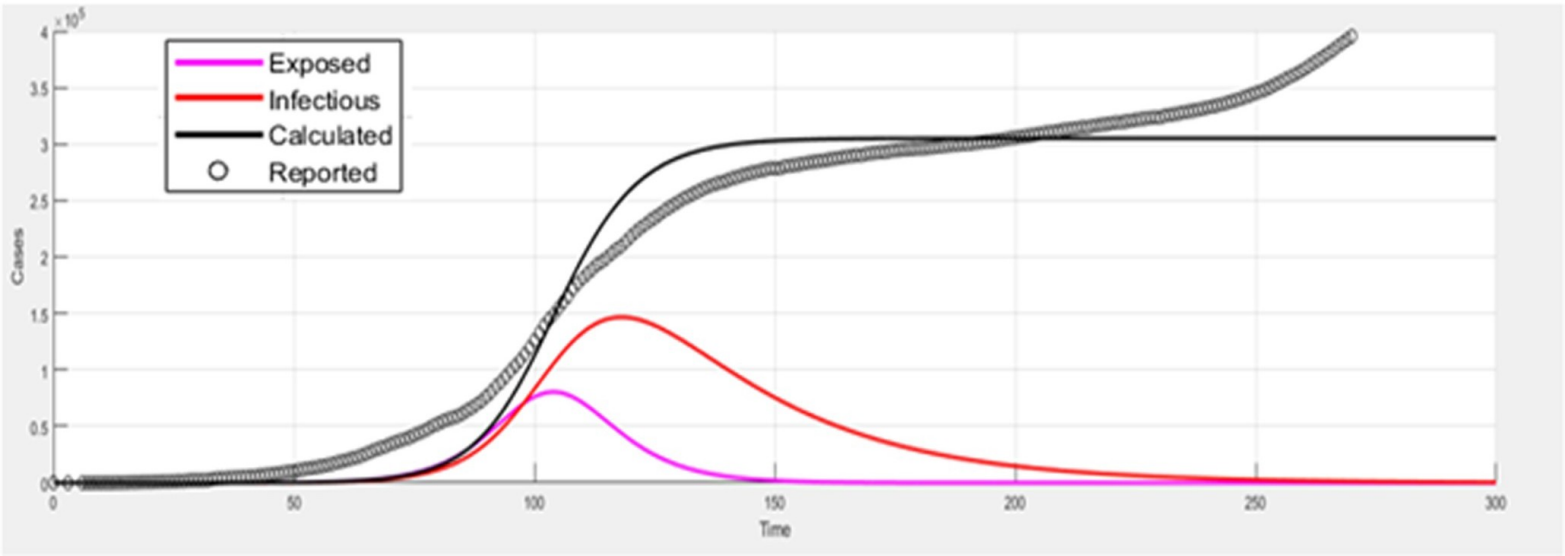
Proposed hybrid model with prediction.

Therefore, this paper initialises the initial healthy number *S*_0_ of the separate SEIR model to 100,000, the exposed person *E*_0_ to 0 and the infected person *I*_0_ to the actual number of infected people on the first day. The recovered person *R*_0_ is initialised to the sum of the number of cured and the number of deaths on the first day. The infection rate β and β_2_ are set according to the SARS infection rate, meaning one person infects 2 to 3 people. Therefore, β and β_2_ are initialised to 3/100000. The infection rate of exposed persons becoming infected is initialised to 2/3, and the recovery rate γ according to the official COVID-19, the cure rate is about 50%, which is initialised to 0.5. The comparison result is shown in Fig 3.

Prediction of the SEIR individual is not satisfactory. Many studies have improved the SEIR model, and to obtain a good prediction effect, seven parameters need to be debugged numerous times, which is a complicated and time-consuming task. In order to understand the prediction effect of the model more clearly, the root mean square error (*RMSE*) and Mean absolute error(MAE) are used for quantitative analysis, as follows:

$$RMSE=\sqrt{\frac{1}{N}\Sigma_{t=1}^{N}{\left(o_{t}-p_{t}\right)}^{2}}$$ (6)

$$MAE=\frac{\Sigma_{i=1}^{n}|y_{i}-x_{i}|}{n}$$ (7)

where *p*_t_ and *o*_t_ represent the *t*-*th* predicted value and true value, and *N* represents the total number of samples.

The analysis results are shown in Table 3 the *RMSE* of the two models differ by nearly 9 times, which further proves the superiority of our hybrid model.

**Table 3:** *RMSE* value of methods.

Models	SEIR	Proposed Hybrid Model
RMSE	31.81	3.67
MAE	3.91	0.98

Compared with the SEIR model, the hybrid model proposed in this paper can significantly reduce the number of tuning parameters when predicting the number of existing diagnoses. At most, three parameters need to be debugged, and no additional data is needed to analyse and initialise the parameters. Regardless of whether it is from the prediction curve or the quantitative analysis of *RMSE*, the proposed model in this paper has shown certain advantages.

### B. Impact of parameters changes

Further, we found that results of this hybrid model change with respect to the parameter changes, such as input population size, growth rate and infection rate.

#### i) *S*_0_ parameter impact

**Fig 4:**
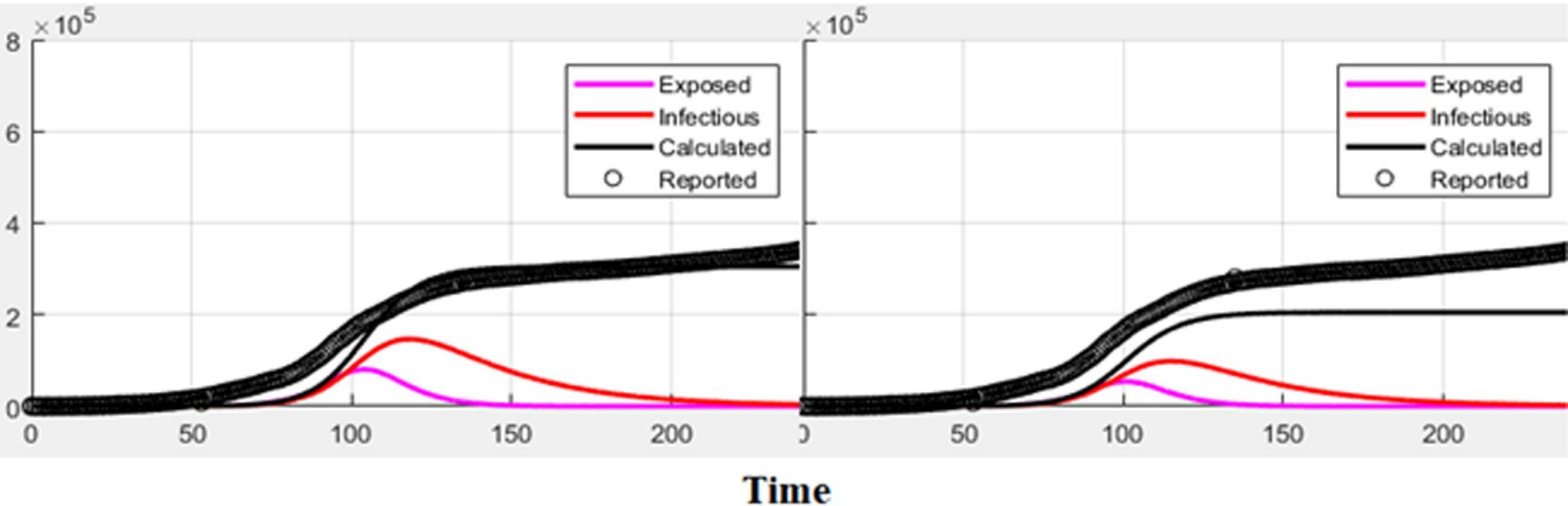
*S*_0_ impact on the results of output.

Analyse the influence of different initial healthy people (*S*_0_) on the prediction results from the prediction curve and weighted error perspective. The prediction curve is shown in Fig 4, and the error is shown in Table 4.

**Table 4:** Impact analysis of population size S_0_.

Rate	RMSE	MAE
K	11.548	1.68
K * 1.10	9.145	0.98
K * 1.30	3.67	1.13
K * 1.60	6.345	1.41

It can be seen intuitively from Fig 4 that the longer the time curve of the various initial values is, the closer the results are, and they all overlap in the end. Therefore, the *S*_0_ parameters that need to be debugged in the proposed model can be debugged relatively easily. This is proven from another aspect. This article proposes the superiority of the model.

From Table 4, when *S*_0_ = *K* × 1.30, the error is the smallest 3.67 and the prediction effect is the best.

#### ii) *α* and *γ* parameter impact

From the perspective of the prediction curve and the error, the influence of different initial values of the conversion rate α from the exposed person to the infected person on the curve prediction effect is studied. Table 5 show the values of the parameter.

**Table 5:** Impact analysis of parameters.

α	RMSE (α)	MAE(α)	γ	RMSE (γ)	MAE(γ)
1	10.11	1.68	1 / 10	9.478	1.61
1/4	3.67	0.98	1 / 14	6.84	1.01
1/8	7.81	1.13	1 / 18	3.67	0.98
1/12	6.345	1.41	1 / 20	4.16	0.99

**Fig 5:**
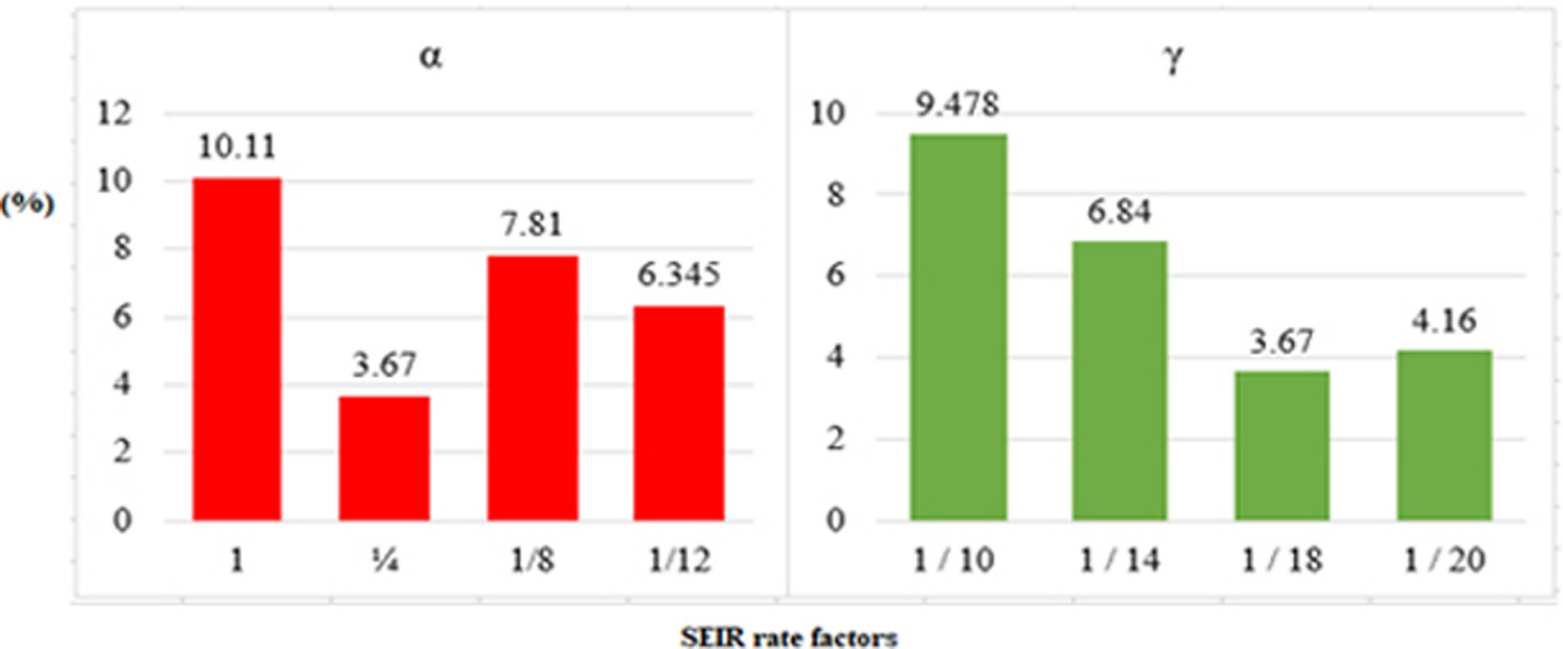
Analysis of different parameters.

Table 5 and Fig 5 show that the weighted error is the smallest when the conversion rate of an exposed person becomes an infected person, α = 1/4, which is 3.67 and has the best effect.

It can be seen from Table 5 that as time increases, the larger the γ, the better the prediction effect. With the development of the epidemic, the cure rate is growing, so as time increases, the prediction effect with a larger γ value will be better. In the actual prediction process, the value of γ can be increased every 4 days on the additional test set (for example, from 1/18, 1/16, 1/14, until it is close to 1). This proves the superiority and good interpretability of this model from the side.

## 5. Discussion

In terms of mathematical models, the SEIR model aims to evaluate infectious diseases. However, the logistic model is designed to change with the evolution of the curve. The logistic model is more compatible with existing data than the SEIR model because it is taught based on existing data. However, it cannot be accurately estimated and contains corruptions. Therefore, we believe that the logistics model is more suitable for forecasting in the future. However, the SEIR model establishes more variables and factors (exposed rate and infectious rate) by considering the interaction and interrelationship of several groups of people. It is more reasonable than the logical model because it follows the rules of infectious disease development, but the prediction results are extensive.

In addition, the proposed model can be improved by adding other classes, and results can be changed if sudden policy implementations are made, such as lockdowns, smart lockdowns and inter-city travel bans. In other models, the benefits may still be sensitive and random. However, it is extremely difficult to find a large amount of missing information and parameter calibration provided by authorised agencies. The model predicts that more people will be infected than the pre-infection and final stages of the epidemic. At the end of the disease, more accurate information about the parameters will be obtained, and complete data can be used to predict the progress of β (and *R*_0_) with time.

**Fig 6:**
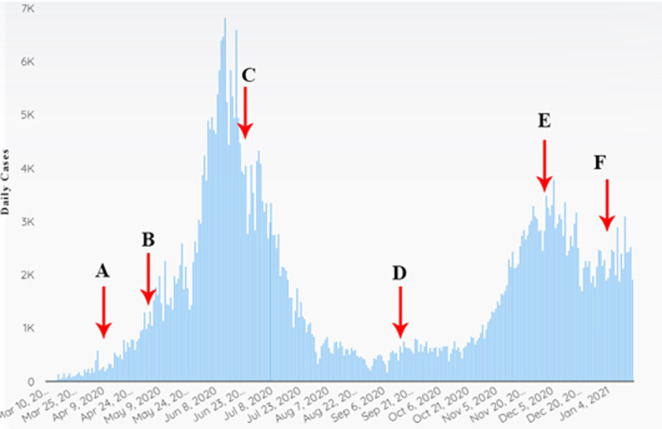
Daily COVID cases in Pakistan.

The outbreak of the pandemic is not only disastrous in terms of casualties but also economically. Therefore, it is important to avoid this situation by taking the necessary steps at the right time. This has not yet been achieved in Italy or around the world. According to these calculations, effective measures are social distancing and family isolation. A health care system that is not designed for normal conditions cannot be prepared for a pandemic. The number of infected people increases exponentially, and disease outbreaks may occur within a few days. If the number of deaths and infectious diseases is high, the number of casualties may be high. The difference in actions taken in just a few days can make a vast difference in preventing this disaster. Fig 6 shows the policy implementations for tackling COVID-19 in Pakistan. At point A, the first lockdown was implemented due to the rise in cases. Despite this increase, the lockdown was lifted at point B. However, educational institutions remained closed from point A. At point C, another lockdown was implemented with some area-wise classification in which the spread was much higher [43]. At point D, the government opened educational institutions again with proper SOP’s and softened the lockdown conditions. This caused the increase in cases until point E. Then, the government shut down the education system until point F. After point F, despite rising COVID cases, education institutes were opened, and again COVID cases peaked. It is much more important to follow proper standards to tackle this disease. China implemented a complete lockdown, low or no travelling for 3 months and mandatory face masks. People in high-risk areas were locked in their apartments, and the government provided support for daily-use consumption items. The proper forecasting and standard implementation of the Chinese government-level policy can help Pakistan deal with this disease quickly.

## 6. Conclusion

With the rise in big data and the development of machine learning, new guidance and ideas have been provided to predict the development of the 2019 coronavirus epidemic. However, machine learning prediction only considers the correlation relationship but does not incorporate the characteristics of the causality relationship. In this study, we combined the SEIR different parameters to extract the final forecasting results based on existing trend of data. Combining the advantages of the two methods and technologies will provide good results and better ideas.

The hybrid model in this paper overcomes the shortcomings of the logistic model’s inability to predict the number of confirmed diagnoses. At the same time, it also solves the problem that, although the SEIR model can predict the number of confirmed diagnoses, it needs to be adjusted based on a large amount of data and requires multiple adjustments. The number of parameters is reduced from 7 to 3. In the part of the experiment, the prediction curve, MAE and *RMSE* proved once again that the proposed model in this paper is superior to the SEIR model alone. This study can be implemented in other countries after availability of data in the required format, this way the model can be helpful in other countries.
